# Non-homogeneous Leukoplakia on the Dorsum of Bifid Tongue: A Case Report

**DOI:** 10.7759/cureus.113181

**Published:** 2026-07-22

**Authors:** Ramachandra Reddy Gowda Venkatesha, Karthik Rajaram Mohan, Saramma Mathew Fenn, Reethikarathan Rathanswamy Thiruneervannan, Sindhuja Rajalingam

**Affiliations:** 1 Oral Medicine and Radiology, Vinayaka Mission's Sankarachariyar Dental College, Vinayaka Mission's Research Foundation (Deemed to be University), Salem, IND

**Keywords:** dorsum of the tongue, er:cr:ysgg laser, er: yag laser, oral leukoplakia, photodynamic therapy (pdt), proliferative verrucous leukoplakia

## Abstract

Leukoplakia does not show clinical characteristics of any other mucosal lesion except for the chronic use of tobacco. Clinical appearance of such lesions displays irregular, discontinuous, or continuous white plaques or non-scrapable patches that may be raised or flat on the surface of the oral mucous membrane. The presence of these lesions necessitates careful clinical and medical evaluation. This clinical image highlights a rare non-homogeneous leukoplakia in a 79-year-old man with a bifid tongue, including clinical features, differential diagnosis, and treatment.

## Introduction

Leukoplakia is a potentially oral premalignant lesion [1]. Leukoplakia clinically appears as a white continuous or homogeneous plaque or non-homogeneous or discontinuous white plaque, white nodular or hair-like elevated proliferative verrucous or speckled forms with red areas [1]. Oral leukoplakia is a white lesion of the oral mucosa that can be described as any other classifiable lesion. Homogeneous leukoplakia presents with a well-demarcated white plaque with a uniform reaction pattern in the whole mucosa [2]. The surface texture ranges from smooth to leathery texture with surface fissures termed as "cracked mud" [2]. Unlike leukoplakia, lichen planus lesions are more cottony and fluffy with no clear demarcation [2]. Lesions of leukoplakia are asymptomatic, whereas oral lichen planus lesions have burning pain in their past history [2,3]. Non-homogeneous leukoplakia is mixed with interspersed patches of red components or erythroplakia or speckled leukoplakia. Nodular leukoplakia consists of a surface covered with nodular, raised, papillary projections [2,3].

## Case presentation

A 79-year-old man reported to the department of oral medicine for a routine dental checkup. The patient's medical history revealed he was not a diabetic, hypertensive, or associated with any other comorbidities. History revealed he had a habit of chewing smokeless tobacco, one packet per day, for the past 15 years. There was no past surgical history for his tongue. There was no history of any associated burning sensation on his tongue. Extraoral examination revealed no pruritic skin lesions. Clinical examination revealed a non-homogeneous white leathery plaque of size measuring 3.5 cm x 3 cm, resembling a mosaic or marble-like appearance on the left lateral dorsum near the tip of the tongue. The right lateral dorsum and lateral borders of the anterior one-third of the tongue were smooth and devoid of papilla intermingled with discrete areas of white plaque and grayish pigmentation. A cleft was observed on the tip of the tongue, resulting in a bifid tongue (Figure 1).

**Figure 1:**
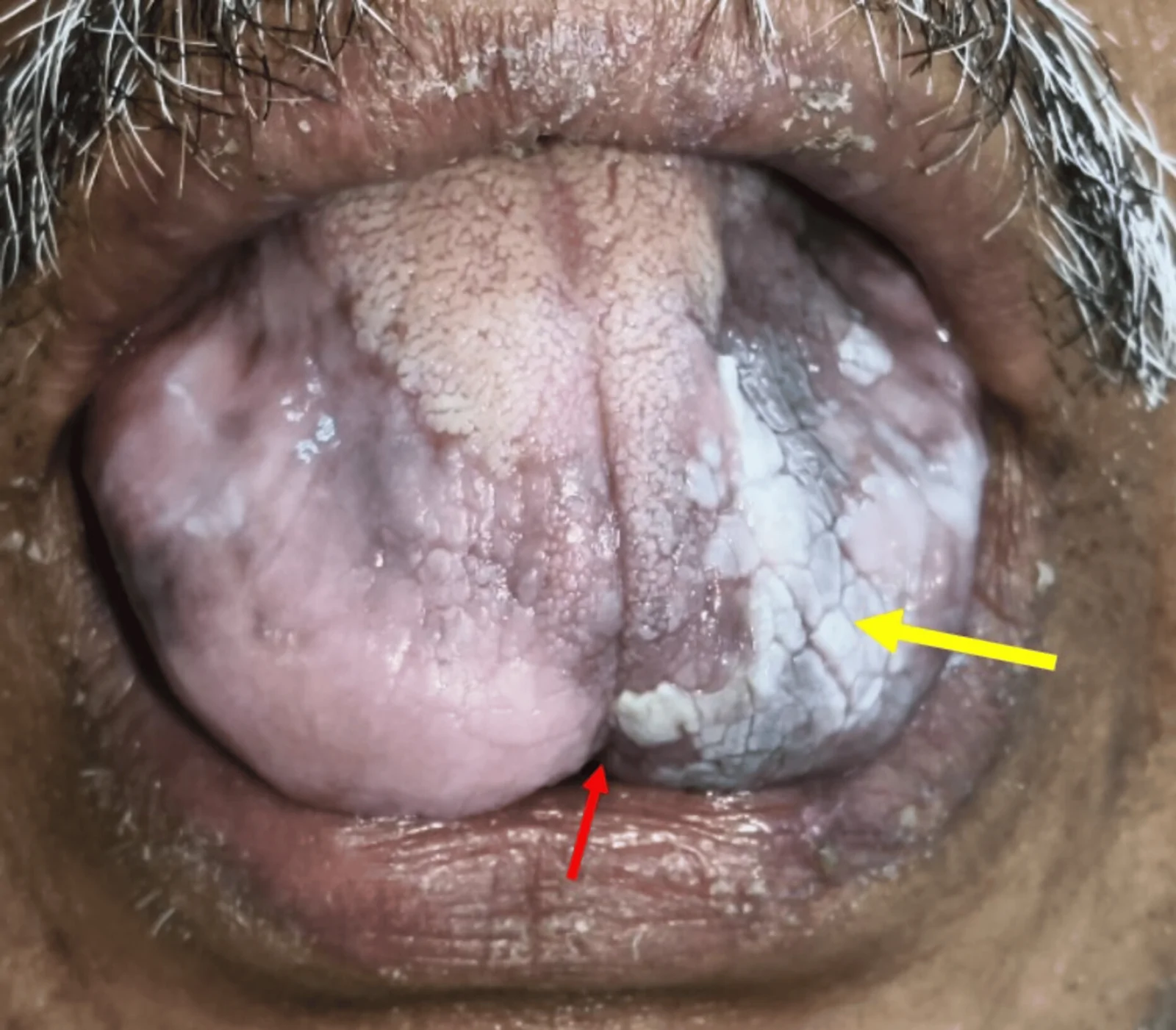
Intraoral examination revealed a non-homogeneous white plaque measuring about 3.5 cm x 3 cm on the left lateral dorsum of the tongue (yellow arrow) and a cleft at the tip of the tongue (red arrow)

There was no restriction in tongue movements. On palpation, it was non-scrapable and non-tender. Based on the above history and clinical findings, a provisional diagnosis of non-homogeneous leukoplakia on the dorsum of the bifid tongue was made. The staging of leukoplakia in our case was L2 PX [1]. Because the size of the leukoplakia was 3.5 cm in our case, and it was designated L2 and PX, dysplasia was not specified. The differential diagnosis was chronic hyperplastic candidiasis, proliferative verrucous leukoplakia, and iron-deficiency anemia. His serum laboratory investigation revealed a hemoglobin percentage of 14.2 g/dL (normal range = 14-17 g/dL). Hence, depapillation of the tongue due to iron-deficiency anemia was ruled out. The patient was followed up after a month, and the white lesion on the left dorsum of the tongue persisted without any associated clinical symptoms such as a burning sensation (Figure 2).

**Figure 2:**
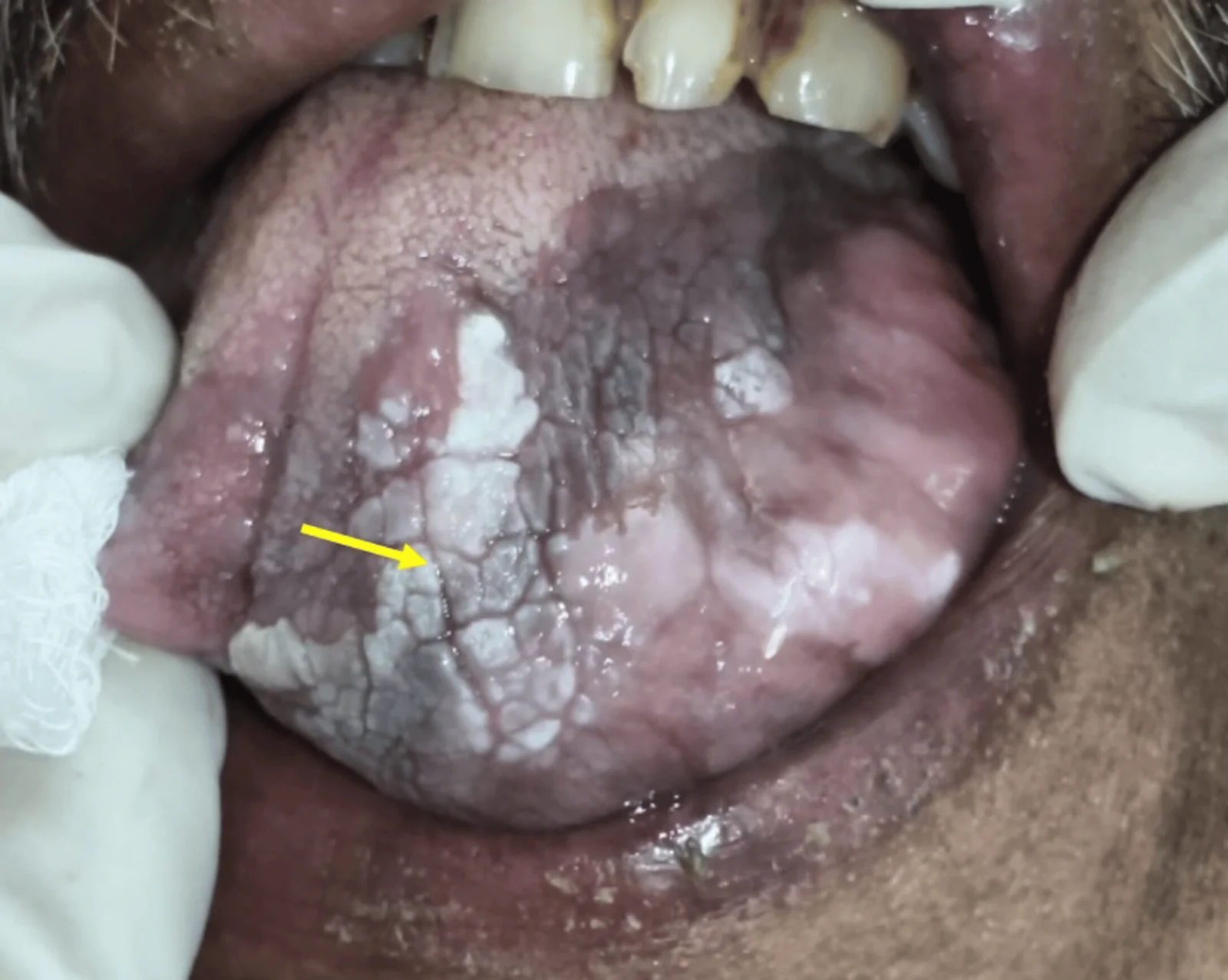
Follow-up after a month revealed a white non-homogeneous plaque on the left dorsum of the tongue (yellow arrow) with no reduction in size of the lesion

Limitations

The patient was completely asymptomatic, with no burning sensation. Hence, he did not consent to the biopsy despite explaining the risk of malignant transformation.

Clinical significance

This case reports a rare, unique occurrence of dorsal tongue leukoplakia and bifid tongue in a 79-year-old man, not reported in any previous literature.

## Discussion

Leukoplakia is of clinical significance due to the presence of dysplasia at some point in development [1]. Oral leukoplakia is the most prevalent form of oral potentially malignant disorder and has been extensively researched [1]. The malignant transformation of oral leukoplakia has always remained a clinical concern. The rate of malignant transformation increases with large size, older age group, location on the tongue, non-homogeneous leukoplakia, and the habit of smoking or smokeless tobacco chewing with or without betel nut [1]. Yang et al. stated that among 144 patients, 44 (25.48%) had leukoplakia lesions on the tongue dorsum [2].

Leukoplakia on the dorsum of the tongue is less commonly observed clinically than leukoplakia on the ventrolateral surface of the tongue. The behavior of leukoplakia on the tongue's dorsum and the tongue's ventrolateral surface concerning carbon dioxide (CO₂) laser treatment is similar in malignant transformation and rate of recurrence. Clinicians must adopt a more aggressive approach to oral tongue leukoplakia with a more severe grade of dysplasia. The cumulative malignant transformation rate is 7.41% [2]. The period of malignant transformation of dorsal tongue leukoplakia is 1.25 to 5.13 years [2].

A bifid tongue, or cleft or forked tongue, is a congenital anomaly that occurs in a small percentage of the population and is characterized by a split or divided tongue, usually resulting from a failure of the embryonic tissues to fuse appropriately during development [4]. A bifid tongue's etiology and related factors are the inadequate fusion of lateral lingual swellings and a median swelling (tuberculum impar) from the first three or four branchial arches during embryogenesis. Inadequate fusion of these structures may lead to a bifid tongue [4]. The bifid tongue occurs with various genetic syndromes, including oral-facial-digital syndrome type I, Opitz syndrome, Klippel-Feil anomaly, Larsen syndrome, and hereditary sensory and autonomic neuropathy type IV [4,5]. Proliferative verrucous leukoplakia is not much attributed to tobacco and alcohol use and has a high mortality, whereas leukoplakia is attributed to tobacco use [5]. Careful monitoring of the lesion is essential, as it is likely to become dysplastic. Patients with a non-homogeneous type of leukoplakia on the tongue develop cancer earlier than those with a homogeneous type. In conclusion, leukoplakia on the tongue dorsum should be closely monitored and potentially treated to avoid future complications. Cessation of risk factors such as tobacco and alcohol consumption and regular dental visits are key preventive strategies.

For non-homogeneous oral leukoplakia, Erbium:Yttrium Aluminium Garnet (Er:YAG), Erbium, Chromium-doped Yttrium, Scandium, Gallium, and Garnet laser (Er:Cr:YSGG), and CO₂ laser with photodynamic therapy (PDT) provide potential alternatives to surgical removal, likely decreasing recurrence and increasing patient comfort [6]. More high-quality randomized controlled trials (RCTs) are needed to verify these results and identify the most appropriate laser-PDT combination for treating oral leukoplakia [6]. More RCTs are required to identify the CO₂ laser as beneficial in treating oral leukoplakia [7,8]. The management for oral leukoplakia includes observation or surveillance, laser or excision by surgery, and local, systemic chemoprevention approaches [9]. A CO₂ laser is used to treat vocal cord leukoplakia and has the advantage of being minimally invasive, having low recurrence post-treatment, and improving phonation after surgery [9]. Laser-assisted or activated photodynamic therapy (LA-PDT) is an effective treatment method for dysplastic oral leukoplakia, which must be treated first with topical aminolevulinic acid, a photosensitizer. Aminolevulinic acid photodynamic therapy (ALA-PDT) uses light-emitting diode (LED) or laser light for a favorable clinical outcome. In addition, ALA-PDT is a non-surgical method that can treat recurrent or refractory lesions without producing significant short- or long-term adverse effects [10].

## Conclusions

A thorough knowledge of the clinical appearance of white lesions on the tongue is essential. A white homogeneous plaque on the dorsum of the tongue needs careful monitoring and assessment, due to the risk of malignant transformation. The advancing older age and clinical increase in the size and unique location on the tongue warrant evaluation. Counselling about cessation of smoking or smokeless tobacco must be enforced forcefully in such affected individuals to prevent the risk of malignant transformation and increase the quality of life.
